# Moral considerability of brain organoids from the perspective of computational architecture

**DOI:** 10.1093/oons/kvae004

**Published:** 2024-03-12

**Authors:** J Lomax Boyd

**Affiliations:** Berman Institute of Bioethics, Johns Hopkins University, 1809 Ashland Ave, Baltimore, MD 21205, USA

**Keywords:** Brain organoids, Cognition, Neuroethics, Moral status, Computational architecture, Information flow, Moral patiency

## Abstract

Human brain organoids equipped with complex cytoarchitecture and closed-loop feedback from virtual environments could provide insights into neural mechanisms underlying cognition. Yet organoids with certain cognitive capacities might also merit moral consideration. A precautionary approach has been proposed to address these ethical concerns by focusing on the epistemological question of whether organoids possess neural structures for morally-relevant capacities that bear resemblance to those found in human brains. Critics challenge this *similarity* approach on philosophical, scientific, and practical grounds but do so without a suitable alternative. Here, I introduce an *architectural* approach that infers the potential for cognitive-like processing in brain organoids based on the pattern of information flow through the system. The kind of computational architecture acquired by an organoid then informs the kind of cognitive capacities that could, theoretically, be supported and empirically investigated. The implications of this approach for the moral considerability of brain organoids are discussed.

## INTRODUCTION

Brain organoids recapitulate many aspects of human neural development [1], and may provide insights into disease pathology [2]. Towards this goal, researchers are generating *in vitro* brain organoids with cell-type diversity [3, 4], areal identity [5–7], vasculature [8], and cytoarchitecture [9–11] that more closely resemble their *in vivo* counterparts, living human brains. These technological advances raise ethical questions about consent of donors [12], biobanking [13], medical and non-medical (e.g. computing) uses [14], commercialization and commodification [15], multi-stakeholder engagement [16], and governance [17, 18]. However, these concerns do not necessarily involve organoids themselves, as potentially sentient or conscious entities with interests that could merit moral consideration [19]^1^. Here, I will focus primarily on the epistemological question of how to determine whether brain organoids have characteristics that ‘exert a moral obligation on us in virtue of the possession of such intrinsic interests’ [21]. Henry Shevelin developed the concept of *psychological moral patiency* as a form of moral status that arises when entities, especially novel and unfamiliar ones (e.g. robots, AI, brain organoids), acquire the kind of cognitive equivalence that contributes to the moral status of more familiar entities (e.g. humans, Great Apes, ect) [21]. Recent scientific discoveries hint at the potential for complex neural activities in brain organoids that are associated with human brains, which has motivated questions of whether brain organoids possess capacities worthy of moral consideration. Yet organoid neural development is severely limited compared to other, even minimally, sentient beings. Some argue that current regulations governing stem cell research are adequate for non-conscious, partially developed, brain organoids [22, 23]. However, recent studies have shown that ‘first generation’ human brain organoids (HBOs), defined as those without significant sensory-cognitive-motor-like feedback systems, are capable of generating spontaneous neural network activities with a likeness to features of human brain function [24, 25]. These data offer preliminary evidence that HBOs, in principle, have the intrinsic neurobiological constituency to support acquisition of more complex cognitive processes. The degree to which HBOs acquire more advanced cognitive-like capacities will help advance neuroscience research, but also inform whether additional ethical oversight is warranted [26].

What capacities might ‘next generation’ HBOs, with significant sensory-cognitive-motor-like feedback, be capable of? Several pioneering efforts from federal governments (e.g. National Science Foundation, BEGIN Organoid Intelligence program), universities (e.g. *The Mind in Vitro* and Organoid Intelligence consortia [27]), and industry (e.g. Cortical Labs) are underway to design neural systems that exhibit synthetic biological intelligence [28]. What kind of framework might we utilize to situate synthetic biological intelligence within our understanding of cognition? Moreover, the approach taken should also inform our ethical stance toward those entities. Similarity-based approaches offer one path by determining whether brain organoids possess neural features that are associated with moral status-conferring cognitive capacities found in humans, namely sentience^2^ and consciousness [29, 31]. Challenges arising from this approach have been discussed [32]. Briefly, they claim that the organization of HBOs are likely to diverge in meaningful ways from standard human neuroanatomy based on methods for inducing differentiation, which subsequently limit the utility of ethical claims based on similarity indicators derived from neural activities embedded within fully developed, neurotypical brains. A viable alternative ought to reflect the range of organoids that can be generated and provide a pathway for inferring cognitive capacities in non-conventional biological entities [33].

Attempts to explain major transitions in cognitive evolution could provide a useful starting point for developing an alternative framework that reflects the apparent plasticity of how neurological systems can be organized. To begin, an evolutionary perspective recognizes the expansive diversity of neural systems in nature that implement cognition and the processes by which these systems acquire novel capabilities [34]. Next, emerging theories in computational neuroscience provide a principled approach for investigating how measurable characteristics, like information flows, contribute to cognitive processes. Taken together, an evolutionary and computational perspective could help situate next generation HBOs within a broader theory of how cognition emerges, and evolves, from the organizing principles of neural systems. Specifically, Barron and colleagues offer a theory to explain major transitions in cognitive evolution that, as I will argue, could be used as a heuristic for determining the epistemological criteria for moral consideration of brain organoids [35]. They speculate that foundational changes in how neural systems control the flow of information were necessary for new cognitive-phenotypic spaces to open up for selection to act on and propagate new cognitive capacities along evolutionary lineages. In their view, the overall computational architecture–defined by the way information flows through the system [36]–is central to making predictions about downstream cognitive potential of an entity or lineage of entities. Here, I introduce an *architectural* approach derived from Barron et al.’s theory that could provide a useful framework for thinking about the epistemological criteria for psychological moral patiency of next generation brain organoids which could acquire the cognitive equivalence of beings with more widely recognized moral status.

First, I briefly describe some of the recent research on cognitive-like neural processes that have been observed in brain organoids. Second, I outline the challenges of similarity-based approaches in ethics discourse. Finally, I describe an architectural-based approach and consider the ethical implications of adopting computational architecture as an epistemological heuristic for neuroethics discourse around brain organoids.

## INFORMATION FLOW IN BRAIN ORGANOIDS

What are the cognitive-like capacities of first generation HBOs? Two studies provide evidence that organoids can self-organize and generate complex neural network dynamics [24, 25]. These observations support Buzsáki’s theory [37] that ‘it is not sensations that teach the brain and build up its circuits. Instead, the brain comes with a preconfigured and self-organized dynamics that constrains how it acts and views the world’. Results from Sharf et al. (2022) seem to support this view, insofar as organoids display neural activities with complex inter-spike dynamics and theta oscillations without external stimulation. But what factors determine the kind of ‘pre-configured and self-organized dynamics’ that brain organoids are capable of, or might be capable of given external stimulation? Individual neurons are theorized to be efficient signal processors [38], while the geometric configuration of neurons has implications for the computational operations of neural networks [39]. These structural features influence how the functional capacity (e.g. total imputed memory capacity) of neuronal networks scale [40, 41]. The internal organization of brain organoids and absolute functional capacity may be two critical factors in shaping the impact of pre-configured dynamics on cognitive traits.

Trujilo et al (2019) observed that neurospheres do not generate the kind of spontaneous nested network activities exhibited by cortical organoids. The authors hypothesize that ‘the spherical arrangement of neurons is insufficient for the emergence of nested oscillations’ [24]. In a separate study, spiking correlations within organoids showed a directional flow of activity driven by some neurons appearing to be highly connected senders or receivers, while others ‘broker’ inputs and outputs [25]. Sharf et al claimed that theta oscillations in brain organoids are similar, partly, to activity patterns that have been observed in the primate neocortex [42], despite lacking evidence of a classic six-layered laminar organization [24, 43]. Efforts to grow organoids with more laminar organizations are being pursued [6, 44] and the impact of these anatomical features on information flow, oscillatory dynamics, and similarity to *in vivo* functional brain activity will inform the architectural approach described below.

What kind of cognitive-like traits might we expect from next generation HBOs? Data to address this question is starting to emerge [27, 45]. Experiments using 2-dimensional (2D) neuronal cultures suggest that surprisingly simple networks can implement computational processes that contribute to cognitive processes. Cultured neurons exhibit a wide range of computational capacities including the ability to perform adaptive signal processing [46, 47], logical operations [39], short-term memory [48]^3^, and blind source separation [49, 50]. The later response type refers to the ability to resolve a mixture of incoming signals into their source components. At the perceptual level, this is known as the cocktail party effect and Isomura et al. found that simple Hebbian plasticity was insufficient to account for blind source separation in neuronal cultures. They provide statistical evidence that such capacities depend on structural rearrangements between neurons. 2D cultures can also adjust their internal activity in a goal-directed manner [30]. Following earlier work [51], Kagan and colleagues developed *DishBrain*, a system that embodies a neuronal network within a virtual environment using electrical feedback to provide external sensory-like feedback for reinforcement learning [30]. The authors claim that simple neuronal cultures can adjust internal activity in a goal-directed manner that minimizes the variational free energy in the system. Moreover, subsequent work showed that neural criticality, a dynamical state thought to be important for attention, learning, and perhaps even consciousness, can also be exhibited by 2D neuronal cultures embodied within virtual environments [52]. Lastly, HBOs were recently shown to efficiently perform unsupervised learning tasks through plasticity-dependent neural network responses [45]. Cai et al (2023) reported that HBOs could perform speech recognition tasks by converting a spatio-temporal pattern of inputs, representing speech data, into electrical outputs that a simple logistic regression model could use to identify individual speakers.

These results demonstrate that computational processes in support of cognition can be implemented by *in vitro* systems. Precautionary discourse aims to determine whether these attributes might also bear cognitive equivalence to indicators of sentience or consciousness observed in humans [22, 29]. Next, I will describe how similarity-based approaches are used to inform precautionary discourse about HBOs.

## CHALLENGES OF SIMILARITY-BASED APPROACHES

Precautionary discourse focuses on addressing the moral status of brain organoids by limiting research if such entities exhibit indicators of sentience or consciousness [29, 53]. The epistemological focus is primarily on the degree of similarity shared between organoids and human brains due, presumptively, to the assumption that if morally-relevant capacities developed in HBOs, then they would most likely be implemented through homologous structures found in adult humans: ‘if an organoid contains structures or mechanisms that any serious and credible theory of the human NCCs [neural correlates of consciousness] posits to be sufficient for conscious experience, we should take proportionate measures to regulate research on that organoid’ [53]. While proponents of the precautionary principle recognize that there is no clear consensus on the definition of consciousness, the relevance of various theories, including Integrated Information Theory (IIT), Global Neuronal Workspace Theory, Temporal Circuit Hypothesis, and others [31, 54], to brain organoid ethics has been considered. However, nearly all of these ‘credible theories’ are based on data from adult, neurotypical, self-reporting humans. Thus, they share common limitations. I will briefly introduce three challenges that limits the usefulness of applying similarity-based indicators to brain organoids^4^. First, there is an incredible amount of variation in the developmental trajectory of organoids due to how they are generated, which can drive their neural features to diverge from those of *in vivo* human brains in ways that undermine similarity-based indicators. Second, the aforementioned technical complication compounds philosophical challenges, such as the Boundary Problem [55], of identifying neural structures for specific cognitive traits, like consciousness, from background patterns of neural activity. Third, the ‘psychological architecture’ of cognitive traits between developmentally and structurally dissimilar entities may not be directly comparable. These divergences could lead to misleading conclusions that are based on similarity, such as instances where consciousness could be falsely assigned when, in fact, there is no consciousness (false positive). Or, we could fail to assign consciousness when, in fact, it is present (false negative) but implemented through nonhomologous neural structures. Diner points out that similarity-based indicators may also be mere epiphenomena of consciousness [56].

Among the various theories of consciousness, the relevance of IIT has received the most attention in the neuroethics literature. The perturbation complexity index (PCI) has been proposed as an empirical indicator of IIT [57] that could be measured in organoids [54]. PCI attempts to capture the extent to which a system can integrate incoming information. This approach is supported by studies conducted in neurotypical adult humans: high PCI scores correlate with fully conscious individuals while lower scores are associated with increasing levels of anesthesia [58]. However, some claim that IIT does not provide a scientific explanation of consciousness [59], and others describe how non-conscious systems (e.g. sediment, or waveforms), could also exhibit positive PCI scores [28]. Recently, cortical cell cultures were found to have positive PCI scores that would indicate non-negligible levels of consciousness. The authors reported that PCI scores in neuronal cell cultures, however, were unchanged after application of neuromodulatory stimulants that were expected to increase PCI responses [60], which led them to conclude that PCI indicators of consciousness likely ‘requires more than activating neuromodulation and that additional factors, such as appropriate circuit architecture, may be necessary’. As an alternative to neural correlates of consciousness, Birch and colleagues (2020) delineate multiple dimensions of consciousness that are more widely distributed and detectable across the animal kingdom [61]. Under this schema, it remains unclear what dimension(s) of consciousness measures like IIT are detecting, which have implications for determining the moral status of nonhuman entities [61, 62]. Niikawa and colleagues argue conscious suffering is important for moral status [26], while Boyd and Lipshitz (2024) describe how distinct dimensions of consciousness are relevant to questions of moral status [26, 63].

While conscious experience is debated as the moral-status-conferring factor [64–66], Birch and Browning (2021) state that ‘[w]e can’t rule out the possibility that sufficiently sophisticated organoids are, or will soon be, sentient: capable of having feelings with a positive or negative quality, such as feelings of pain or pleasure. If they are sentient, then there are moral limits on what we can do to them’ [53]. The authors then suggest several welfare indicators of sentience (e.g. brain region identity, telomere length, hippocampal volume, neurotransmitter release) that could be identified in HBOs [29]. Brain regions for valence processing are most often associated with sentience, yet challenges remain [29]. First, while data suggest the existence of hedonic hotspots in adult human brains, other regions are likely involved [67]. Which regions, and what degree of similarity, should we base inferences about the capacity of brain organoids to have valenced experiences? Furthermore, if such a list and threshold could be established, how should these criteria be applied to organoids with neural systems that vary in size, structure, neural identity, and physiological activity across batches, lines, and protocols? Second, valenced processing is widely distributed across the entire adult brain [68, 69]. The tendency to over-ascribe cognitive or psychological states, such as valence, to particular brain regions emphasizes several assumptions (the localization, one-to-one, and independence) that have recently been criticized based on accumulating evidence about brain-behavior relationships [70]. Third, HBOs with closed-loop interactions with digital systems are biological-silicon hybrids [45] and these organoids may acquire cognitive-computational capacities that are based, in part, on interactions between digital and biological components that make inferences of any cognitive capacity based on neuroanatomy tenuous. Finally, the concept of ‘emotional valence’, which has long been associated with the amygdala, is increasingly being viewed from a computational perspective that could be implemented by non-amygdala structures [71]. The shift toward computational thinking may serve as a more generalizable pathway for exploring cognitive potential in next generation HBOs.

Ideally, an alternative to similarity-based approaches would be i) *interpretable*, meaning that assessments of brain organoids capacities are based on principled or theoretical frameworks of how cognition is implemented in biological systems, ii) *adaptable*, it affords flexibility for neural systems organized in non-conventional ways, and iii) it should be *relevant* to the ascertainment of cognitive capacities that are epistemologically informative for moral consideration. Next, I introduce Barron *et al’*s theory of cognitive evolution and provide some initial impressions on how their theory can be adapted as an alternative epistemological heuristic to similarity-based approaches.

## AN ARCHITECTURAL VIEW OF INFORMATION FLOWS AND COGNITIVE POTENTIAL

Evolved nervous systems enable adaptive responses to the environment. As such, the biological processes that implement cognition have undergone significant reorganizations across evolutionary time. Barron and colleagues propose a theory to explain these major transitions, which they argue ‘should be identified [through] structural changes in the systems that implement cognition’ [35]. The authors propose five ‘computational architectures’ that are defined by how basic operations, representations, memory, and control flows are managed [36]. Control flow is particularly important, the authors claim, because it determines how different parts of the nervous system coordinate, share information, and influence one another. ‘Hence changes in control flow are important for determining what specific cognitive capacities are evolvable’ once a new architecture has been established [35]. Evidence for any given architecture should provide clues as to what kinds of cognitive processes an organoid might be able to implement under appropriate experimental conditions. I argue that this computational-based taxonomy of cognition affords us an opportunity to design an *interpretable*, *adaptable*, and *relevant* model for anticipating the kind of cognitive capacities, and moral considerations, that next generation HBO might soon exhibit.

### Five computational architectures of information flow

**Decentralized architectures** are found in unicellular and multicellular organisms, such as the hydra and jellyfish, and are likely the antecedents of more complex networks. They can be based on chemical signaling or distributed neuronal networks, and work to control information flow through local interactions and effectors. In this context, information flow is highly regionalized and capable of supporting cognitive-like functions related to habituation, sensitization, and basic forms of non-associative learning that underlie behavioral responses to the environment. Decentralized networks can also be functionally organized by modality, such as the subnetworks responsible for distinct behaviors in hydras [72]. The capacities of decentralized networks share some resemblance to the organization and response dynamics of 2D neuronal cultures capable of basic computations (and positive PCI scores) described earlier.

Nematodes are representatives of **centralized architectures** with nervous systems that have a ‘master controller’ to help coordinate global, animal-wide responses to sensory inputs and homeostatic states [73]. These feed-forward-dominated systems share similarities with convolutional neural networks where information can be integrated across layers. Compared to decentralized nervous systems, these architectures support multimodal learning, cross-modal recall, context learning, and latent inhibition. Theoretically, the directional information flow observed in brain organoids [25] could support responses in a closed-loop virtual environment [25] that resemble animals with centralized architectures. Next, the evolution of **recurrent architectures** in insects, for example, enabled more abstract forms of information processing to be implemented. The integration of feedback and feedforward loops allows information to reverberate within networks and influence earlier processes throughout the system. This allows for forms of working memory, selective attention, error prediction, and perhaps basic modeling of future events. So far, data analyzed from brain organoids have not reported these patterns of information flow, but future experiments or analysis could reveal that HBOs exhibit such patterns of information flow [74].

The **laminated architectures** of mammalian brains are composed of multiple systems that function, in parallel, with many other subsystems through recurrent looping interactions. Barron *et al* clarify that laminated architectures are also found in avian brains with pallial structures that are organized into nuclei, rather than cytoarchitectural layers, as the term might imply. In mammalian and avian brains, information flows are recurrent between cortical/pallial regions and subcortical, midbrain, and cerebellar-like subsystems. Neural ensembles responsible for different capacities can process information in parallel with varying temporal and spatial scales. This enables ‘the abstraction of information, operating with sequences of information or the production of complex sequences of behavior, and forward modeling of the consequences of action’ [35]. The same information can be multiplexed for distinct purposes, including valence processing, memory retrieval, association, and motor coordination. As brains with laminated architectures scale in size, such as the expansion of the frontal cortices during human origins, the functional capacity of these networks rapidly expands. Brain organoids currently lack indicators of cytoarchitectural layering or laminated information flows. Even next generation HBOs that do achieve some degree of layered cytoarchitecture will be limited in capacity by allometric constraints over nutrient availability [75].

The final transition toward **reflective architecture** enabled adaptive, intentional, and perhaps conscious adjustment of information flow. Here, control flow is less constrained by physical structures, but rather, virtual control flows that can be redirected by downstream information processing in a manner similar to self-writing code in computer science. Networks with reflective architectures can iterate over the query, *‘what is the best control flow for this task?*’, and adjust the kind of information processing that is applied in a particular context. Barron et al. speculate that this transition, along with brain scaling, was necessary for the emergence of symbolic language [76], which were prerequisites for ‘collaborative computation’ that occurs across individuals and communities. Even next generation HBOs are expected to lack these architectures and resulting cognitive capabilities for the foreseeable future. But an understanding of how these architectures implement cognition will be an important component of developing next generation HBOs that model aspects of human neurocomputational processes.

### Implications of computational architecture for moral considerability

What are the ethical implications of adopting computational architecture? It seems reasonable to assume that brain organoids are unlikely to acquire the full functional capacity of adult human brains in the immediate future. Should brain organoids start to exhibit the cognitive equivalence, or pattern of information flow, associated with human-like cognition (e.g virtual or laminated architectures) then serious ethical and moral consideration may be warranted. More realistically, brain organoids will incrementally acquire functional sophistications that signal the acquisition of increasingly structured information flows. Given the uncertainty of how this technology might develop, an approach that can generate hypotheses about the relationship between form and function would provide a useful heuristic for determining the epistemological criteria that constitutes cognitive equivalence between brain organoids and sentient beings. The ability to generate empirically-testable hypotheses would help address the kind of uncertainties that are inherent to brain organoids, and stem cell technologies, more generally. The *Stem Cell Uncertainty Principle* describes how two conjugated parameters–functional potential and actualized phenotype–cannot be determined at the same time [77]. Empirical indicators of computational architecture enable us to map out the kind of cognitive phenotypes that could arise from next generation HBOs.

**Figure 1:**
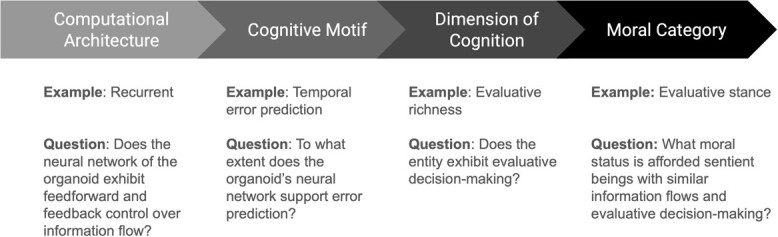
**An epistemological heuristic for moral considerability.** A conceptual flowchart, from left to right, is provided that describes how stages of scientific inquiry can lead toward moral consideration. Results from each stage, which are informed by empirical evidence from the previous stage, can be used as a heuristic for determining the kinds of experimental studies and moral considerations that should be applied toward brain organoids

The epistemological frame of computational architecture enables us to better understand how cognitive capacities are ‘built up’ from principled components, like information flows. Empirical evidence for specific kinds of control flows generate hypotheses about the kinds of cognitive capacities that could, theoretically, be exhibited. From here, we can lay out an experimental workflow that moves from descriptions of basic nervous system organization and information flow toward evidence of cognitive capacities that could merit moral consideration (Fig. 1). Indicators of computational architecture may, but not necessarily, signal the possession of cognitive motifs that may, or not, support the implementation of morally-relevant dimensions of cognition. Each step along the path informs the kinds of cognitive capacities that could be elicited under appropriate conditions. A subset of which are informative for determining moral status, but also for the scientific study of non-moral-status-conferring capacities that advance our basic understanding of cognition. For neuroethical considerations, it would be helpful to have some way of relating cognitive capacities to moral status.

Boyd and Lipshitz offer one option by connecting specific dimensions of cognition with criteria for moral status [63]. They ground four cognitive capacities associated with consciousness (evaluative richness, unity, temporality, and self-awareness) [61] with features that contribute to full moral status identified by philosopher Stefan Gosepath (evaluative stance, agency, self-directedness, other-directedness) [78]. For example, conscious self-awareness matters, morally, because it enables the expression of agency, self-directedness, and directedness toward other beings. This dimension of consciousness likely requires a reflective architecture that supports the kinds of collaborative cognition described earlier. Unity and temporality are important to moral status because they contribute to self-directedness, or the ability to have a concept of self with interests that can endure (or be deprived of) over time. Laminated architectures, and possibly sophisticated versions of recurrent architectures, are likely needed to support memory recollection, forward modeling, and action selection needed to maintain an enduring unified concept of self. Information processing that can occur in parallel enables neural systems to make evaluations that incorporate past knowledge, current homeostatic states, future events, and the outcomes of action selection–all of which are likely necessary for capacities that support agency and self-directedness. Evaluative richness represents a dimension of consciousness most closely aligned with how ethicists view the role of sentience in welfare assessments. While decentralized and centralized architectures can support adaptive nociceptive-like responses, the emotional state of *suffering* or *frustration* may require mechanisms for associative learning in a context-dependent manner that integrates internal feedback across several subsystems that establish an expectation for non-suffering under specific conditions [79]. I hypothesize that the capacity to have an evaluative stance, or to be sentient from the perspective of ethicists, may require neural circuit motifs found in recurrent architectures in order for the entity to calculate the temporal or reward-based error predictions that inform, together with other subsystems, whether the entity ought be frustrated or upset given some internal state or external context [71, 80]. Importantly, the link between computational architecture and specific cognitive capacities are yet to be confirmed by data, but nonetheless, are capable of generating experimentally tractable hypotheses.

**Figure 2:**
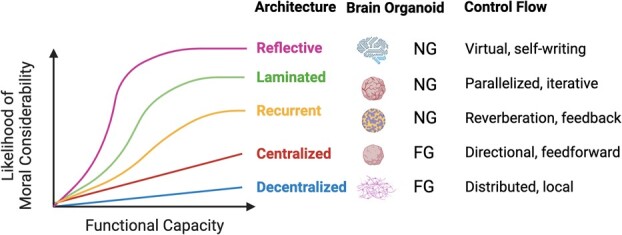
**The predicted impact of computational architecture on moral considerability.** The moral status, or moral considerability, of first generation (FG) and next generation (NG) brain organoids are hypothesized to be a function of the computational architecture and overall capacity for memory storage, information representation, parallelization, or other functional capacities

A case to consider: should an organoid with recurrent architecture and evidence of an evaluative stance merit moral consideration? And if yes, then how much? While Boyd and Lipshitz argue that certain capacities contribute to moral status, the actual degree of moral consideration assigned to any particular dimension of consciousness, or entity that possesses them, is left open. Under the architectural framework, ethical considerations begin with collecting data that differentiates between computational architectures, followed by determining conditions for evaluating overlaying cognitive motif structures and more specific capacities, and finally, assessing the overall functional capacity of the system using an appropriate behavioral or input-response experimental design, as described in more detail by Boyd and Lipshitz (2023). Altogether, I expect the likelihood of moral considerability to be architectural-specific (Fig. 2) based on evidence that describes how functional capacities scale across different networks. Memory capacities are important for conscious dimensions of unity and temporality, for instance. They are estimated to scale faster in humans, who have virtual architectures, compared to rodents with more laminated architectures, due to species-specific differences in axonal morphology that impact the rate of growth in network complexity [41]. Encephalization of cortical areas in humans would be expected to amplify these scaling differences in functional output. Under the proposed schema, an organoid with recurrent architecture would merit some non-negligible moral (status) consideration should evidence of a meaningful evaluative stance, for example, also be demonstrated (Fig. 1). Overall, architectural features may constrain the upper limit of moral consideration, or likelihood of moral consideration, based on the expectant cognitive potential enabled by specific patterns of information flow. Entities with distributed architectures, for example, are predicted to not support the kind of information flows that implement morally-relevant capacities achievable in laminated systems (but other ethical considerations for these entities may be warranted, such as relational or ecological value of the entity under consideration).

The nature of the relationship between architecture and amount of moral considerability represents a future area of scholarship for experimental neuroethics, but also affords two practical benefits for assigning moral status. First, the architectural approach affords the opportunity to compare the information flows of brain organoids to those of animals with similar patterns of control, who may already possess a recognized moral status. These comparisons of cognitive equivalence are useful for advancing organoids as scientific models of cognitive function, in general, but also providing insights into the neurobiological pathways that contribute to the moral status of sentient beings. Second, the incremental nature of the architectural approach affords an opportunity for participants in deliberative governance to weigh the risks and benefits of brain organoid systems. Civil society has the chance to consider how brain organoid technology should be used given a set of civic priorities, perceived risks, expressed values, and ontological beliefs that inform the rationale for developing next generation brain organoids with potentially non-negligible moral status, for medical, commercial, or other applications [18].

## COUNTER ARGUMENTS

There are several counterarguments to consider. First, critics may contend that the architectural approach is a reformulation of similarity-based approaches derived from a different set of indicators. However, the similarity indicators used in precautionary discourse are based on correlated measures (e.g. PCI) associated with morally-relevant traits (e.g. consciousness) in humans. The architectural approach is a heuristic organized around computational principles that are more hypothesis-generating in nature. For example, if an organoid exhibits architecture *x*, then cognitive capacity *y* is predicted, and the relationship itself is the subject of study. A second counterargument to consider is whether future research will reveal that any given architecture, which was thought to *not* support neurocomputational processes important for moral status-conferring cognitive capacities can, in fact, do so. This is a reasonable critique given that Barron et al.’s theory of cognitive evolution is the product of our limited understanding of how biological systems implement cognition. Under this scenario, the relationship between information flow (e.g. architecture) and cognitive capacity would need to be revised, as data becomes available, thereby providing a heuristic for mapping the pathway toward moral considerability (Fig. 1).

## CONCLUSIONS

The task of how to assign a specific computational architecture to any particular brain organoid is left open. Efforts to measure the functional connectome of brain organoids using multielectrode arrays, calcium imaging, or other histological methods could provide empirical indicators of how information flows within these systems. A number of whole-animal or brain region connectomes are becoming available in numerous species that will enable more direct comparisons with brain organoids. Moreover, the development of analytic tools based on graph theory are providing new methods for quantifying the functional connectomes of biological neural networks [81, 82]. Finally, the identification of specific neural circuit motifs that contribute to each computational architecture will be a necessary first step toward determining the epistemological criteria that might be appropriate for moral considerations of next generation brain organoids, and other non-conventional beings.

## DATA AVAILABILITY

No new data was generated for this study.

## STUDY FUNDING and APC FUNDING

JLB is supported by grants from the Kavli Foundation, the Johns Hopkins University Whiting School of Engineering and Applied Physics Laboratory (SURPASS).

## AUTHORS' CONTRIBUTIONS

 JLB (Conceptualization-Equal, Funding acquisition-Equal, Investigation-Equal, Visualization-Equal, Writing – original draft-Equal).

## DISCLOSURES

The author has no relevant financial or non-financial interests to disclose.

## Supplementary Material

Web_Material_kvae004

## Footnotes

1 Here, I will use the term ‘moral considerability’ and ‘moral status’ interchangeably to mean an entity whose wellbeing and interests should be taken into consideration [20].

2 The ethics and neuroscience literatures use the term ‘sentience’ to describe conceptually distinct capacities. For ethicists, sentience often means the capacity to feel pain or pleasure, or the ability to have valenced experiences (see [29], for example). Alternatively, Kagan et al. (2023) define sentience as ‘responsive to environmental information’ [30].

3 Neural ‘memories’ lasting less than one second are thought to depend upon recurrent neuronal architectures

4 See [32] for more detailed discussion.
