# Supplementary material for: Moral considerability of brain organoids from the perspective of computational architecture
Source: Oxf Open Neurosci. 2024 Mar 12;3:kvae004. doi: 10.1093/oons/kvae004 (PMC10995847; doi:10.1093/oons/kvae004)
Supplement: Web_Material_kvae004 [file web_material_kvae004.docx]

**REVIEWER COMMENTS**

**OXFNSC-2023-018 - Computational architecture shapes cognitive potential in next generation brain organoids: implications for neuroethics**

**Original submission**

**Reviewer 1**

Comments to the Author

Thank you for the opportunity to review the manuscript titled "Computational Architecture Shapes Cognitive Potential in Next Generation Brain Organoids: Implications for Neuroethics." This manuscript offers a fascinating exploration of the ethical dimensions surrounding the development of brain organoids. However, I believe certain revisions are necessary to further enhance its clarity, depth, and overall scholarly impact.

Major Comments:

1. The introduction section would benefit from additional context regarding the current research landscape in brain organoids, particularly focusing on their ethical implications. Providing a concise overview of recent advancements in this domain would lay a stronger foundation for the subsequent discussion.

2. Currently, the literature review appears somewhat biased towards specific facets of brain organoid development and ethical considerations. I recommend broadening this review to encompass a diverse range of viewpoints and debates within this sphere. Such a comprehensive approach would significantly augment the manuscript's depth and breadth.

3. Addressing Counterarguments: The manuscript would gain considerable strength by incorporating a dedicated section that addresses possible counterarguments or critiques. This inclusion would not only exhibit a thorough grasp of the subject matter but also fortify the overall argument presented.

Implementing these suggested revisions should substantially enhance the manuscript's contribution to the ongoing discourse on brain organoids and neuroethics.

**Reviewer 2**

Comments to the Author

The paper 'Computational architecture shapes cognitive potential in next generation brain organoids: implications for neuroethics' addresses a particularly relevant topic in the recent biomedical literature and does so with an original ethical approach worthy of consideration.

The paper is mainly based on some recent research (Baron et al., 2023; Sharf et al., 2022), which is certainly interesting and promising. But this should suggest more caution *to the authors* when presenting their theses. This means emphasising more in the paper that as far as the information flow in the brain and brain organoids is concerned, we are still at a stage of early research and data to be confirmed.

Above all, it would be useful, in my opinion, to explain in more detail, perhaps in the Conclusions section, what the ethical implications might be of adopting computational architecture as a physiological basis for assessing the state of consciousness and the cognitive level and, consequently, the degree of moral status of human brain organoids.

Minor points

The author might at least briefly consider two very recent papers that analysed the main theories of consciousness in relation to human brain organoids, https://doi.org/10.1080/21507740.2023.2173329 ; DOI: 10.1017/S0963180123000543

I think it would be better to exclude ref 44, as the attack in question on IIT is not scientific in nature but related to aspects of communication and scientific and academic rivalries, which should not be mixed with evidence-based research.

**Decision letter**

15-Nov-2023

Dear Prof. Boyd,

Manuscript ID OXFNSC-2023-018 entitled "Computational architecture shapes cognitive potential in next generation brain organoids: implications for neuroethics" which you submitted to the Oxford Open Neuroscience, has been reviewed. The comments of the reviewer(s) are included at the bottom of this letter.

The manuscript has been reviewed by two experts in the field, and while finding your manuscript interesting, indicated that several changes should be made.

Please note that this journal operates with transparent peer review. This means that if your submission is accepted for publication, the full peer review history of your article will publish online alongside your article. This includes reviewer comments, editor decision letters, and your author responses.

The reviewer(s) have recommended publication, but also suggest some major revisions to your manuscript. Therefore, I invite you to respond to the reviewer(s)' comments and revise your manuscript within the next 60 days from today date.

To revise your manuscript, log into https://mc.manuscriptcentral.com/oxfnsc and enter your Author Centre, where you will find your manuscript title listed under "Manuscripts with Decisions." Under "Actions," click on "Create a Revision." Your manuscript number has been appended to denote a revision.

You may also click the below link to start the revision process (or continue the process if you have already started your revision) for your manuscript. If you use the below link you will not be required to login to ScholarOne Manuscripts.

*** PLEASE NOTE: This is a two-step process. After clicking on the link, you will be directed to a webpage to confirm. ***

https://mc.manuscriptcentral.com/oxfnsc?URL_MASK=f4edb3e0aab547b1b59c36404140d43d

You will be unable to make your revisions on the originally submitted version of the manuscript. Instead, revise your manuscript using a word processing program and save it on your computer. Please also highlight the changes to your manuscript within the document by using the track changes mode in MS Word or by using bold or colored text.

Once the revised manuscript is prepared, you can upload it and submit it through your Author Centre.

When submitting your revised manuscript, you will be able to respond to the comments made by the reviewer(s) in the space provided. You can use this space to document any changes you make to the original manuscript. In order to expedite the processing of the revised manuscript, please be as specific as possible in your response to the reviewer(s).

IMPORTANT: Your original files are available to you when you upload your revised manuscript. Please delete any redundant files before completing the submission.

Because we are trying to facilitate timely publication of manuscripts submitted to the Oxford Open Neuroscience, your revised manuscript should be uploaded within the next 60 days from today date. If it is not possible for you to submit your revision in a reasonable amount of time, we may have to consider your paper as a new submission.

Once again, thank you for submitting your manuscript to the Oxford Open Neuroscience and I look forward to receiving your revision.

Sincerely,

Dr. Orly Reiner

Senior Editor, Oxford Open Neuroscience

orly.reiner@weizmann.ac.il, orly.reiner@weizmann.ac.il

Reviewer: 1

Comments to the Author

Thank you for the opportunity to review the manuscript titled "Computational Architecture Shapes Cognitive Potential in Next Generation Brain Organoids: Implications for Neuroethics." This manuscript offers a fascinating exploration of the ethical dimensions surrounding the development of brain organoids. However, I believe certain revisions are necessary to further enhance its clarity, depth, and overall scholarly impact.

Major Comments:

1. The introduction section would benefit from additional context regarding the current research landscape in brain organoids, particularly focusing on their ethical implications. Providing a concise overview of recent advancements in this domain would lay a stronger foundation for the subsequent discussion.

2. Currently, the literature review appears somewhat biased towards specific facets of brain organoid development and ethical considerations. I recommend broadening this review to encompass a diverse range of viewpoints and debates within this sphere. Such a comprehensive approach would significantly augment the manuscript's depth and breadth.

3. Addressing Counterarguments: The manuscript would gain considerable strength by incorporating a dedicated section that addresses possible counterarguments or critiques. This inclusion would not only exhibit a thorough grasp of the subject matter but also fortify the overall argument presented.

Implementing these suggested revisions should substantially enhance the manuscript's contribution to the ongoing discourse on brain organoids and neuroethics.

Reviewer: 2

Comments to the Author

The paper 'Computational architecture shapes cognitive potential in next generation brain organoids: implications for neuroethics' addresses a particularly relevant topic in the recent biomedical literature and does so with an original ethical approach worthy of consideration.

The paper is mainly based on some recent research (Baron et al., 2023; Sharf et al., 2022), which is certainly interesting and promising. But this should suggest more caution *to the authors* when presenting their theses. This means emphasising more in the paper that as far as the information flow in the brain and brain organoids is concerned, we are still at a stage of early research and data to be confirmed.

Above all, it would be useful, in my opinion, to explain in more detail, perhaps in the Conclusions section, what the ethical implications might be of adopting computational architecture as a physiological basis for assessing the state of consciousness and the cognitive level and, consequently, the degree of moral status of human brain organoids.

Minor points

The author might at least briefly consider two very recent papers that analysed the main theories of consciousness in relation to human brain organoids, https://doi.org/10.1080/21507740.2023.2173329 ; DOI: 10.1017/S0963180123000543

I think it would be better to exclude ref 44, as the attack in question on IIT is not scientific in nature but related to aspects of communication and scientific and academic rivalries, which should not be mixed with evidence-based research.

**Author response**

Dear Editor and Peer Reviewers:

The comments and suggestions provided to the author were helpful in honing the arguments made in this Brief Perspective article on the development of an architectural approach to understanding cognitive potential and moral considerability of human brain organoids. Significant changes were made throughout the manuscript with the exception of the section describing the computational architectures and background of information flows reported for organoids, where only minor changes or additions were implemented.

In response to Reviewer 1: The author has made a more concerted effort in the introduction section to situate the ethical issue discussed in this Brief Perspective, which focus on the moral status of organoids, within the larger scope of ethical concerns that arise from methodological advances in brain organoid technology. The author mentions consent of donors[12], biobanking[13], medical and non-medical (e.g. computing) uses[14], commercialization and commodification[15], multi-stakeholder engagement[16], and governance[17,18] with relevant references. The author has also expanded discussion of viewpoints and debates that are relevant to the moral status of brain organoids in the ‘Challenges of similarity-based approaches’ section with specific mention of points made by Diner (2023) . Also, various theories of consciousness, including Integrated Information Theory (IIT), Global Neuronal Workspace Theory, Temporal Circuit Hypothesis, and others[29, 53] are mentioned with subsequent focus on ITT which has received the most attention in ethics discourse. As suggested, the author has added several counterarguments to consider in the ‘Conclusions’ section of the manuscript.

Review 2 responses: The author recognizes the importance of emphasizing the preliminary nature of the Barron et al theory of major transitions in cognitive evolution. The author has added qualifying text throughout the manuscript that calls attention to this fact. Moreover, additional language has been added in appropriate sections to emphasize more clearly that an architectural-based approach calls for experimental validation, which results could also inform the revision of underlying assumptions regarding the relationship between information flows and specific cognitive capacities. Figure 1 was added to illustrate the conceptual flow of experimental studies that could be applied to organoids. The authors made significant changes in the section ‘Implications of an architectural approach for moral considerability’ to explore the implications that result from adopting information flows as a scaffold for understanding brain organoid cognitive-like capacities with adjustments to Figure 2 to illustrate the relationship between architecture and moral considerability. The reviewer’s recommendation to include, and exclude, specific references have been reflected in the manuscript.

**OXFNSC-2023-018.R1 - A computational view of cognitive potential and moral considerability of brain organoids**

**Reviewer 1**

Comment to the Author

The paper offers several intriguing insights. Notably, the authors introduce a fresh perspective on the functional assessment of human brain organoids and associated ethical considerations. However, I have a suggestion concerning the paper's structure: the positioning of the counterarguments of an architectural-based approach in the conclusion seems somewhat awkward. I propose relocating this section to either stand alone before the conclusion or be integrated into the preceding section for better coherence.

**Reviewer 2**

Comments to the Author

Note 5 should be reformulated as the references are cited by numbers and not by publication year

Please control lines 53-56 on p. 9 and 3-4 on p. 10 for grammar and repetition of "based"

**Decision letter**

29-Dec-2023

Dear Prof. Boyd,

Manuscript ID OXFNSC-2023-018.R1 entitled "A computational view of cognitive potential and moral considerability of brain organoids" which you submitted to the Oxford Open Neuroscience, has been reviewed. The comments of the reviewer(s) are included at the bottom of this letter.

Please note that this journal operates with transparent peer review. This means that if your submission is accepted for publication, the full peer review history of your article will publish online alongside your article. This includes reviewer comments, editor decision letters, and your author responses.

The reviewer(s) have recommended publication, but also suggest some minor revisions to your manuscript. Therefore, I invite you to respond to the reviewer(s)' comments and revise your manuscript within the next 30 days from todays date.

To revise your manuscript, log into https://mc.manuscriptcentral.com/oxfnsc and enter your Author Centre, where you will find your manuscript title listed under "Manuscripts with Decisions." Under "Actions," click on "Create a Revision." Your manuscript number has been appended to denote a revision.

You may also click the below link to start the revision process (or continue the process if you have already started your revision) for your manuscript. If you use the below link you will not be required to login to ScholarOne Manuscripts.

*** PLEASE NOTE: This is a two-step process. After clicking on the link, you will be directed to a webpage to confirm. ***

https://mc.manuscriptcentral.com/oxfnsc?URL_MASK=61f7a8964c354c2d946a7b8fb46e7da0

You will be unable to make your revisions on the originally submitted version of the manuscript. Instead, revise your manuscript using a word processing program and save it on your computer. Please also highlight the changes to your manuscript within the document by using the track changes mode in MS Word or by using bold or colored text.

Once the revised manuscript is prepared, you can upload it and submit it through your Author Centre.

When submitting your revised manuscript, you will be able to respond to the comments made by the reviewer(s) in the space provided. You can use this space to document any changes you make to the original manuscript. In order to expedite the processing of the revised manuscript, please be as specific as possible in your response to the reviewer(s).

IMPORTANT: Your original files are available to you when you upload your revised manuscript. Please delete any redundant files before completing the submission.

Because we are trying to facilitate timely publication of manuscripts submitted to the Oxford Open Neuroscience, your revised manuscript should be uploaded within the next 30 days from todays date. If it is not possible for you to submit your revision in a reasonable amount of time, we may have to consider your paper as a new submission.

Once again, thank you for submitting your manuscript to the Oxford Open Neuroscience and I look forward to receiving your revision.

Sincerely,

Dr. Orly Reiner

Senior Editor, Oxford Open Neuroscience

orly.reiner@weizmann.ac.il, orly.reiner@weizmann.ac.il

Reviewer: 2

Comments to the Author

Note 5 should be reformulated as the references are cited by numbers and not by publication year

Please control lines 53-56 on p. 9 and 3-4 on p. 10 for grammar and repetition of "based"

Reviewer: 1

Comments to the Author

The paper offers several intriguing insights. Notably, the authors introduce a fresh perspective on the functional assessment of human brain organoids and associated ethical considerations. However, I have a suggestion concerning the paper's structure: the positioning of the counter arguments of an architectural-based approach in the conclusion seems somewhat awkward. I propose relocating this section to either stand alone before the conclusion or be integrated into the preceding section for better coherence.

Associate Editor

Comments to the Author:

(There are no comments.)

**Author response**

The reference style for Note 5 has been reformatted to the style used in the remainder of the manuscript. The grammatical suggestions on lines 53-56 on p. 9 and 3-4 on p. 10 for grammar and repetition of "based" have been incorporated. The paragraph exploring several counter arguments have been relocated to a separate section, as suggested by one of the reviewers.

Moreover, small edits were made throughout several sections of the manuscript to increase clarify and readability of the concepts. The section addressing neuroethical implications of the architectural approach received additional edits that further clarified the how the framework impacts the assessment of moral status in brain organoids.

**OXFNSC-2023-018.R2 - A neurocomputational view of cognitive potential and moral considerability of brain organoids**

**Reviewer 1**

Comments to the Author

The manuscript is structured around a detailed examination of current and potential future capabilities of human brain organoids (HBOs), grounded in recent empirical studies and theoretical advances. It explores the implications of these capabilities for moral considerability, suggesting that as organoids increasingly mirror complex neural activities, they may warrant additional ethical oversight. The discussion extends to the implications of organoid research for understanding cognition, sentience, and consciousness, proposing that an architectural-based approach could facilitate a more nuanced understanding of the moral status of brain organoids.

While the manuscript makes a significant contribution to the ongoing ethical debate surrounding brain organoid research and proposes an innovative framework for evaluating the moral status of increasingly complex in vitro neural models, two key areas require attention:

On page 7, lines 38-41, the phrase "If they do" could potentially lead to confusion about what it refers to. A more explicit way to articulate this might be, "Should brain organoids develop capacities that warrant moral consideration...". However, this section may require further elaboration, as the paper does not extensively discuss the concept of full moral status or the criteria determining such status.

The concept of moral patiency, mentioned on page 2, line 32, requires clarification for readers who may not be familiar with the term.

Addressing these two points will strengthen the manuscript's clarity and comprehensiveness, further solidifying its contribution to the field.

**Reviewer 2**

Comments to the Author

The author has successfully addressed the comments I raised. I take the paper to be publishable in the current form.

**Decision letter**

03-Feb-2024

Dear Prof. Boyd,

Manuscript ID OXFNSC-2023-018.R2 entitled "A neurocomputational view of cognitive potential and moral considerability of brain organoids" which you submitted to the Oxford Open Neuroscience, has been reviewed. The comments of the reviewer(s) are included at the bottom of this letter.

Please note that this journal operates with transparent peer review. This means that if your submission is accepted for publication, the full peer review history of your article will publish online alongside your article. This includes reviewer comments, editor decision letters, and your author responses.

The reviewer(s) have recommended publication, but also suggest some minor revisions to your manuscript. Therefore, I invite you to respond to the reviewer(s)' comments and revise your manuscript within the next 30 days from todays date.

To revise your manuscript, log into https://mc.manuscriptcentral.com/oxfnsc and enter your Author Centre, where you will find your manuscript title listed under "Manuscripts with Decisions." Under "Actions," click on "Create a Revision." Your manuscript number has been appended to denote a revision.

You may also click the below link to start the revision process (or continue the process if you have already started your revision) for your manuscript. If you use the below link you will not be required to login to ScholarOne Manuscripts.

*** PLEASE NOTE: This is a two-step process. After clicking on the link, you will be directed to a webpage to confirm. ***

https://mc.manuscriptcentral.com/oxfnsc?URL_MASK=6c0a5e227aa943ed942d41fa3528978a

You will be unable to make your revisions on the originally submitted version of the manuscript. Instead, revise your manuscript using a word processing program and save it on your computer. Please also highlight the changes to your manuscript within the document by using the track changes mode in MS Word or by using bold or colored text.

Once the revised manuscript is prepared, you can upload it and submit it through your Author Centre.

When submitting your revised manuscript, you will be able to respond to the comments made by the reviewer(s) in the space provided. You can use this space to document any changes you make to the original manuscript. In order to expedite the processing of the revised manuscript, please be as specific as possible in your response to the reviewer(s).

IMPORTANT: Your original files are available to you when you upload your revised manuscript. Please delete any redundant files before completing the submission.

Because we are trying to facilitate timely publication of manuscripts submitted to the Oxford Open Neuroscience, your revised manuscript should be uploaded within the next 30 days from todays date. If it is not possible for you to submit your revision in a reasonable amount of time, we may have to consider your paper as a new submission.

Once again, thank you for submitting your manuscript to the Oxford Open Neuroscience and I look forward to receiving your revision.

Sincerely,

Dr. Orly Reiner

Senior Editor, Oxford Open Neuroscience

orly.reiner@weizmann.ac.il, orly.reiner@weizmann.ac.il

Reviewer: 2

Comments to the Author

The author has successfully addressed the comments I raised

I take the paper to be publishable in the current form

Reviewer: 1

Comments to the Author

The manuscript is structured around a detailed examination of current and potential future capabilities of human brain organoids (HBOs), grounded in recent empirical studies and theoretical advances. It explores the implications of these capabilities for moral considerability, suggesting that as organoids increasingly mirror complex neural activities, they may warrant additional ethical oversight. The discussion extends to the implications of organoid research for understanding cognition, sentience, and consciousness, proposing that an architectural-based approach could facilitate a more nuanced understanding of the moral status of brain organoids.

While the manuscript makes a significant contribution to the ongoing ethical debate surrounding brain organoid research and proposes an innovative framework for evaluating the moral status of increasingly complex in vitro neural models, two key areas require attention:

On page 7, lines 38-41, the phrase "If they do" could potentially lead to confusion about what it refers to. A more explicit way to articulate this might be, "Should brain organoids develop capacities that warrant moral consideration...". However, this section may require further elaboration, as the paper does not extensively discuss the concept of full moral status or the criteria determining such status.

The concept of moral patiency, mentioned on page 2, line 32, requires clarification for readers who may not be familiar with the term.

Addressing these two points will strengthen the manuscript's clarity and comprehensiveness, further solidifying its contribution to the field.

Associate Editor

Comments to the Author:

Please revise according to the comments made by reviewer #1.

**Author response**

The author would like to thank Reviewer 1 for highlighting two issues that require further clarification. In regards to the first concern clarifying the sentence starting with 'If they do ..." , the author has revised the text to focus on how the architectural approach is envisioned as an epistemological heuristic for identifying cognitive capacities that could lead to further moral consideration (and perhaps moral status).

"Should brain organoids start to exhibit the cognitive equivalence, or pattern of information flow, associated with human-like cognition (e.g virtual or laminated architectures) then serious ethical and moral consideration may be warranted. More realistically, brain organoids will incrementally acquire functional sophistications that signal the acquisition of increasingly structured information flows. Given the uncertainty of how this technology might develop, an approach that can generate hypotheses about the relationship between form and function would provide a useful heuristic for determining the epistemological criteria that constitutes cognitive equivalence between brain organoids and sentient beings."

The author has added additional explanatory text for moral patiency at the introduction, clarified the role of this perspective in the overall aims of the manuscript, and added text throughout the manuscript that connects back to the concept of patiency based on cognitive equivalence.

"However, these concerns do not necessarily involve organoids themselves, as potentially sentient or conscious entities with interests that could merit moral consideration[19] . Here, I will focus primarily on the epistemological question of how to determine whether brain organoids have characteristics that ‘exert a moral obligation on us in virtue of the possession of such intrinsic interests’[21]. Henry Shevelin developed the concept of psychological moral patiency as a form of moral status that arises when entities, especially novel and unfamiliar ones (e.g. robots, AI, brain organoids), acquire the kind of cognitive equivalence that contributes to the moral status of more familiar entities (e.g. humans, Great Apes, and others)[21]. Questions of whether brain organoids possess these moral status-conferring cognitive capacities has been motivated by recent scientific discoveries that hint at the potential for complex neural activities that are observed or associated with human brains."

**OXFNSC-2023-018.R3 - Moral considerability of brain organoids from the perspective of computational architecture**

**Reviewer 1**

Comments to the Author

I would like to express my gratitude for your detailed and thoughtful response to my comments on your manuscript titled "A neurocomputational view of cognitive potential and moral considerability of brain organoids." Your revisions and the additional clarifications provided have effectively addressed the concerns I raised. Given the comprehensive revisions and the quality of the discourse presented, I believe that this manuscript is indeed worthy of publication. Thank you for the opportunity to review this work and for your diligent attention to the review comments.

**Decision letter**

27-Feb-2024

Dear Prof. Boyd,

It is a pleasure to accept your revised manuscript entitled "Moral considerability of brain organoids from the perspective of computational architecture" in its current form for publication in the Oxford Open Neuroscience. The comments of the reviewer(s) who reviewed your manuscript are included at the foot of this letter.

Please note that this journal operates with transparent peer review. This means that the full peer review history of your article will publish online alongside your article. This includes reviewer comments, editor decision letters, and your author responses.

Next steps

You will receive an email from no-reply@scipris.com within roughly one week. This is your invitation to sign up for an account with SciPris, Oxford University Press’ author portal hosted by Aptara. You will need to create an account if you do not already hold one. Please register or log into your account and follow the online instructions which will guide you through signing your licence and paying the APC. The email and the portal have clearly signposted support options if you need any help during this process.

Please note that SciPris is a completely different system from ScholarOne, so your credentials to submit your manuscript here will not work there. Once you’ve created a SciPris account, you will be able to use it whenever you publish with Oxford Open Neuroscience or any OUP journal. Please note that OUP will only ever request payment for applicable fees be made via SciPris or to an OUP bank account. If you ever have concerns about the legitimacy of a request, please do not hesitate to contact a customer services agent via the SciPris portal or directly via oupsupport@scipris.com.

Thank you for your fine contribution. On behalf of the Editors of the Oxford Open Neuroscience, we look forward to your continued contributions to the Journal.

Sincerely,

Dr. Orly Reiner

Senior Editor, Oxford Open Neuroscience

orly.reiner@weizmann.ac.il, orly.reiner@weizmann.ac.il

Reviewer: 1

Comments to the Author

I would like to express my gratitude for your detailed and thoughtful response to my comments on your manuscript titled "A neurocomputational view of cognitive potential and moral considerability of brain organoids." Your revisions and the additional clarifications provided have effectively addressed the concerns I raised. Given the comprehensive revisions and the quality of the discourse presented, I believe that this manuscript is indeed worthy of publication. Thank you for the opportunity to review this work and for your diligent attention to the review comments.

Associate Editor

Comments to the Author:

(There are no comments.)
